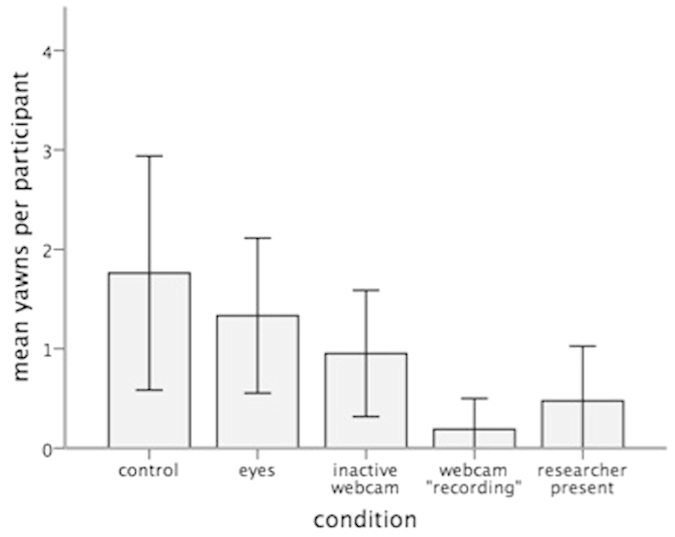# Social Presence Diminishes Contagious Yawning in the Laboratory

**DOI:** 10.1038/srep25045

**Published:** 2016-04-26

**Authors:** Andrew Gallup, Allyson M. Church, Heather Miller, Evan F. Risko, Alan Kingstone

**Affiliations:** 1 Psychology Department, State University of New York at Oneonta, Oneonta, New York 13820, USA; 2 Department of Psychology, University of Waterloo, Ontario, Canada N2L 3G1; 3 Department of Psychology, University of British Columbia, Vancouver, BC Canada V6T 1Z4.

## Abstract

Contagious yawning may be a useful measure of social psychological functioning, and thus it is important to evaluate the variables influencing its expression in laboratory settings. Previous research has documented that humans yawn less frequently in crowded environments and when under direct observation, but the impact of social presence on contagious yawning remains unknown. Here we present the first study to systematically alter the degree of social presence experienced by participants in the laboratory to determine its effect on contagious yawning frequency. Our results demonstrate that both implied and actual social presence significantly diminish yawn contagion in comparison to a control condition, indicating a key social component to contagious yawning. These findings provide a framework for pursuing additional research investigating the social factors influencing contagious yawning, while also offering applications for measuring this response in laboratory settings.

Yawning is characterized by a powerful gaping of the jaw with inspiration, a brief period of peak muscle contraction, and a passive closure of the jaw with shorter expiration^1^. Although seemingly indistinguishable in the motor pattern, yawns are elicited in two distinct ways. Unlike spontaneous yawning, which is triggered physiologically perhaps due to modified arousal or state changes associated with brain temperature fluctuations^2^,^3^,^4^, contagious yawning is psychologically driven by sensing or thinking about the action. While spontaneous yawning appears to be widespread among vertebrates^5^, thus far only humans and a limited number of non-human species have been documented to yawn contagiously^6^,^7^,^8^,^9^,^10^,^11^,^12^,^13^.

Interest in contagious yawning has grown substantially in recent years, following studies linking individual differences in this response to various measures of perspective taking and empathic processing^14^,^15^. The development of this empathic modeling hypothesis has led to research using contagious yawning as a potential dependent measure for assessing empathy in both typically developing and clinical populations. Most recently, it has been reported that individuals scoring higher on a psychopathic personality inventory showed reduced contagious yawning^16^. Initial reports on the absence of contagious yawning in children with autism spectrum disorder also supported this connection to empathy^17^,^18^,^19^, but subsequent research shows that this result may be a consequence of the reduced tendency for these individuals to attend to others’ faces^20^,^21^. In addition, at least one study has failed to demonstrate a correlation between empathy and contagious yawning in healthy populations^22^. A positive connection between contagious yawning and social closeness/affiliation has been reported in a number of studies^8^,^9^,^10^,^23^,^24^,^25^,^26^,^27^,^28^, though it appears to be age-dependent (i.e., juveniles fail to show this effect)^29^,^30^ and at least three studies have failed to demonstrate this relationship in adult populations^13^,^31^,^32^. Consequently, the link between contagious yawning and empathy requires further investigation. Nonetheless, the use of contagious yawning could still prove useful for studying social psychological development if a clear social link was established.

Previous research has demonstrated that a variety of behavioral responses in both humans and non-human animals can be modulated as a function of social presence, a phenomenon sometimes known as the audience effect^33^,^34^,^35^. Furthermore, under brief periods of social crowding, human and non-human primates often limit forms of social interaction (i.e., the elevator effect)^36^. To date, the social variables influencing the expression of contagious yawning in humans are relatively unknown. Although previous research has reported that yawning frequency is less common among humans in naturally crowded environments^5^ and in participants being observed by a researcher in the laboratory^37^, the impact of social presence on contagious yawning has yet to be formally investigated. Here we present the first study to systematically alter the type of social presence experienced by participants in the laboratory to determine its effect on contagious yawning frequency.

One of the defining attributes of research on social presence effects is the wide array of stimuli, other than actual people, that have been demonstrated to induce it (e.g., images of eyes^38^,^39^; video cameras^40^,^41^; eye trackers^42^,^43^). This has led to a critical distinction between two broad types of social presence effects: implied social presence effects and actual social presence effects. In the current experiment we investigate the social component to contagious yawning by presenting stimuli that varied in their degree of social presence. In particular, we compared contagious yawning in an “alone” condition with that behavior in the presence of an image of eyes, a webcam without instructions that it was recording, a webcam with instructions that it was recording, and, finally, an actual person. Thus, the former two manipulations (i.e., eyes, webcam + no instructions) merely suggest that the participant is being watched, whereas in the latter two (i.e., webcam with instruction, real person) this notion is made more explicit. In addition, in the case of the latter two manipulations, where the notion that one is being watched is made explicit, we can contrast an implied presence (i.e., a recording webcam) directly with an actual social presence (i.e., a real person). How contagious yawning varies (if at all) across these different types of social presence will provide novel insight into the influence and sensitivity of contagious yawning to the presence of others.

## Results

**Figure 1:**
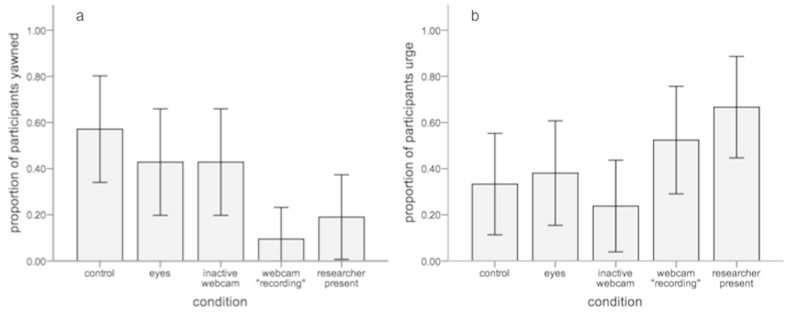
The self-reported occurrence of (**a**) yawning and (**b**) the urge to yawn without doing so per condition (95% CI).

A total of 36 participants reported yawning during the entire experiment. There was no difference in the proportion of males and females that reported yawning contagiously across conditions (*X*^2^(1) = 0.190, *p* = 0.663). Results show that contagious yawning was modulated by social presence (*X*^2^(4) = 14.118, *p* = 0.007; Fig. 1a). Most yawning occurred during the control condition (57%), followed by the eyes and inactive webcam conditions (42.9% in each), and then the researcher present (19.0%) and webcam ‘recording’ (9.5%) conditions. Corrected post-hoc tests showed contagious yawning was lower in both the webcam ‘recording’ and researcher present conditions in comparison to the control condition (i.e., alone with no social presence) (*p* = 0.010; *p* = 0.035). In addition, contagious yawning was lower in the webcam ‘recording’ condition in comparison to both the eyes and inactive webcam conditions (*p* = 0.035; *p* = 0.035). The unsatisfied urge to yawn also varied across conditions (*X*^2^(4) = 9.722, *p* = 0.045; Fig. 1b). The reported urge to yawn without doing so was highest in the researcher present (66.7%) and webcam ‘recording’ (55.0%) conditions, followed by the eyes (38.1%), control (33.3%) and inactive webcam conditions (23.8%). Corrected post-hoc tests revealed that the urge to yawn without doing so was more common in the researcher present condition in comparison to the inactive webcam condition (*p* = 0.050). No other comparisons were significant.

There was also no difference in the number of contagious yawns reported between males and females (*Z* = 0.606, *p* = 0.544). An investigation into yawning frequency revealed a similar main effect of social presence (*X*^2^(4) = 13.842, *p* = 0.008; Fig. 2). Corrected post-hoc tests showed that on average the number of contagious yawns was lower in the webcam ‘recording’ condition than the control (*p* = 0.010) eyes (*p* = 0.040) and inactive webcam (*p* = 0.040) conditions. In addition, contagious yawning frequency was lower in the researcher present condition in comparison to the control condition (*p* = 0.040). No other comparisons were significant.

## Discussion

Overall these findings demonstrate that both implied and actual social presence diminishes contagious yawning in a laboratory setting, and in doing so thus suggest that the social environment plays a key role in the expression of this behavior. Although the specific mechanism(s) producing these effects were not under investigation, we believe there are two likely candidates. First, since yawning has been linked with enhanced cortical arousal^2^, the feeling of being watched could increase levels of arousal to a point that would counteract mechanisms normally triggering yawning. Second, the social stigma associated with displaying this behavior in the presence of others^44^ could lead to the active inhibition of yawn contagion. The increased urge to yawn without doing so in conditions with greater social presence supports the latter interpretation, but follow-up research could attempt to disentangle the physiological and psychological mechanisms contributing to this effect.

With respect to our comparisons between different types of social presence manipulations, the results clearly demonstrated the importance of making the putative “observation” explicit (i.e., there was little effect of eyes or an inactive webcam) and, interestingly, that once the observation is explicit that there was effectively no difference between an implied and actual social presence (i.e., an active webcam and an actual person), at least with respect to the suppression of contagious yawning. The latter effect conceptually replicates earlier research^40^ and extends it to a new domain. One potential explanation for this pattern of results is that the influence of a social presence is contingent on the conscious activation in working memory of the idea that one is being observed. With an explicit implied social presence (i.e., a recording webcam, CCTV) and an actual person present, this activation is direct whereas in the case of eyes and an inactive webcam this idea would require that the individual spontaneously make an inference to that effect.

The view that the influence of a social presence requires conscious activation of the idea that one is being observed draws some support from recent research investigating eye trackers as an implied social presence. Specifically, Nasiopoulus *et al*.^42^ demonstrated that eye tracker induced social presence effects^43^ disappeared if participants wore the eye tracker for a short time before the critical part of the experiment but that it reappeared if, right before that part of the experiment, participants were reminded about the eye tracker. One interpretation of this result is that participants in the former condition, over time, had “forgotten” that they were being observed (i.e., the idea was no longer active in working memory) and that in the latter condition the reminder reactivated this idea. Whether a similar pattern would be observed with an actual social presence remains an open question for future research.

In conclusion, this investigation provides a clear link between social presence and contagious yawning, and as such has direct applications for the design of future studies utilizing contagious yawning as a dependent variable^45^. In particular, since observation cues and recording devices inhibit contagious yawning, future research should control for these factors when seeking to maximize this response. Furthermore, the present study lays the groundwork for further investigations of the effect of social factors on contagious yawning in humans. The current findings support the validity of previous research that has suggested connections with contagious yawning and measures of social cognition, meaning that future studies could explore how individual differences in empathic processing and personality relate to this response. Considering humans display a familiarity bias for contagious yawning (i.e., people yawn more frequently following yawns of friends and family members in comparison to yawns of acquaintances and strangers)^24^, follow-up studies could also investigate whether the inhibitory social presence effects that we observed in the present study are modulated by the nature of interpersonal relationships.

## Methods

A total of 105 (79 female) college-aged participants (19.12 ± 4.54 yrs) were recruited through the psychology pool at a public four-year college in upstate New York during fall 2014. The experiment was carried out in accordance with approved human ethics guidelines, and all participants provided informed consent prior to partaking in this study. The Institutional Review Board at the State University of New York at Oneonta approved this research (#2014–65).

Upon arriving at the laboratory participants were escorted into an individual testing room and instructed to watch and pay close attention to a 170 sec contagious yawning stimulus video on a computer monitor (taken from Platek *et al*.^14^). This stimulus included a randomized and continuous display of ~7 sec video clips of people either yawning, laughing or with neutral expressions, which has been shown to induce contagious yawning in roughly half of all participants in an experimental setting^14^,^46^. Since the rate of spontaneous yawning is quite infrequent^5^,^47^,^48^,^49^, and studies have demonstrated that participants yawn much more frequently when watching a video stimulus depicting yawns compared to control stimuli^50^, we can be confident that the vast majority of yawns reported in the current experiment were contagious. Participants were randomly assigned to one of five conditions while watching the stimulus: (1) alone in the room (control group); (2) alone with a picture of a person’s eyes positioned on top of the monitor; (3) alone in the room with an inactive webcam positioned on top of the monitor; (4) alone in the room with a webcam positioned on top of the monitor that they were told was active and would be recording them during the experiment (although in reality it was inactive); (5) with a researcher sitting behind them and facing in their direction during the presentation of the video. Afterwards participants completed a survey indicating their age, time of testing, hours of sleep the night before, and whether they yawned or if they had the urge to yawn during the experiment. Previous research has demonstrated self-report as a valid measure of yawning in laboratory settings^51^. Furthermore two recent studies have confirmed these findings by revealing substantial agreement between self-report and video confirmed yawns during experimentation (yes/no: Cohen’s *k* = 0.631–0.670; total: Cohen’s *k* = 0.511)^52^,^53^.

The proportion and frequency of yawning was compared between males and females using a chi-square test and a Mann-Whitney test, respectively. Generalized Linear Mixed Models were used to simultaneously examine the effect of social presence condition, time of testing (range: 0900–1515 h), participant age, and hours of sleep the previous night (7.433 ± 1.252 h) on the occurrence of contagious yawning and the urge to yawn without doing so during the experiment (binary logistic). However, testing time, age, and hours of sleep were not significant predictors, and the best model (based on Corrected Akaike Information Criteria) included only the fixed effect of social presence. Therefore chi-square tests were used to compare the proportion of yawners and those that experienced the urge without doing so across social presence conditions. In addition, a Generalized Linear Mixed Model was used to investigate the effects of these variables on yawning frequency. Similar to above, the best model included only the fixed effect of social presence and therefore a Kruskal-Wallis test was used to compare yawning frequency across social presence conditions. Multiple test corrections were applied to all post-hoc comparisons^54^.

## Additional Information

**How to cite this article**: Gallup, A. *et al*. Social Presence Diminishes Contagious Yawning in the Laboratory. *Sci. Rep.* **6**, 25045; doi: 10.1038/srep25045 (2016).

## Figures and Tables

**Figure 2:**